# Dermoid Cyst in the Posterior Fossa Associated With Dermal Sinus and Abscess Formation: A Case Report

**DOI:** 10.7759/cureus.38032

**Published:** 2023-04-23

**Authors:** Basem Bahakeem, Taif F Alqahtani, Abdullah Darwish, Alaa Alkhotani, Elham Rawah

**Affiliations:** 1 Department of Neuroradiology, Radiology Section, Umm Al-Qura University, Makkah, SAU; 2 Department of Internal Medicine, Umm Al-Qura University, Makkah, SAU; 3 College of Medicine, Umm Al-Qura University, Makkah, SAU; 4 Department of Radiology, Neuroimaging Section, King Abdullah Medical City, Makkah, SAU; 5 Department of Pathology, Umm Al-Qura University, Makkah, SAU

**Keywords:** case report, abscess, posterior fossa, cerebellum, dermoid cyst

## Abstract

Posterior fossa dermoid cysts are rare intracranial tumors. Most are congenital and develop during early pregnancy but manifest later in life. We report a case of a congenital posterior fossa dermoid cyst in a 22-year-old patient presenting with fever and multiple neurological complaints. Imaging studies revealed a bony defect in the occipital bone suggestive of sinus formation, heterogeneous hypointensity on T1-weighted image (T1WI), and post-contrast peripheral enhancement suggestive of an infectious process and abscess formation. The histopathological examination was typical for a dermoid cyst containing adnexal structures. This report reviews the case with its unique location and unusual radiological features. Further, the clinical presentation, diagnosis modalities, and treatment outcomes are discussed.

## Introduction

Dermoid cysts originate from a combination of ectodermal and mesodermal tissue during the third to fifth week of pregnancy. They are benign growths, but malignant transformation can happen in rare cases. Although they can occur on any part of the body, they are more commonly found on the head and neck [1-3].

The majority of dermoid cysts are present at birth and typically arise during early development. About 60% of cases are identified in children aged five years or younger, with approximately 40% being diagnosed at birth. However, dermoid cysts can also be acquired following surgery or accidental trauma. This can result in the enclosure and incorporation of ectodermal tissue [4].

Dermoid cysts within the brain itself (intra-axial) are uncommon as intracranial tumors, accounting for only 0.25% to 1.9% of cases. Among them, those found in the posterior fossa are the rarest, ranging from 0.05% to 0.5%. Dermoid cysts are typically found in the subtentorial space near the parasellar region, rather than solely in the posterior fossa, which is an unusual location [5,6].

On rare occasions, the development of a dermoid cyst may extend beyond the confines of the brain and involve the occipital bone and skin surrounding it. This may result in a dermal sinus, a narrow channel lined with an epithelial layer containing skin glandular structures. A dermal sinus that persists can create a pathway for microorganisms to enter the intracranial cyst, increasing the risk of abscess formation [7,8].

Cerebellar abscesses with dermoid cysts in posterior fossa-associated dermal sinus are infrequent, with only 14 cases been reported and an estimated incidence rate of around 14% [9]. This is thought to be due to the shedding of epithelial cells and glandular secretions within the cyst [10].

Fortunately, modern neuroradiological techniques have made the diagnosis of intracranial lesions easier and also allow for more effective surgical planning. However, a preoperative concern arises when a dermoid cyst presents as an intra-axial tumor.

## Case presentation

A 22-year-old female patient with a history of cerebral palsy, epilepsy, and congenital hydrocephalus presented with a two-week history of fever of unknown origin, proceeded by aspiration pneumonia from a seizure attack. The patient was compliant with antiepileptic drugs for 12 years but stopped with full recovery. During her pneumonia, the patient also experienced headaches and vomiting. She had a right frontal ventriculoperitoneal (VP) shunt placed since birth to treat her congenital hydrocephalus, which stopped functioning after 12 years. A new VP shunt was subsequently inserted. There was no history of reduced level of consciousness, photophobia, or stiffness in the neck.

She was then admitted for two weeks to the referring hospital and received antibiotics (cefepime, metronidazole, and vancomycin) with no improvement in her neurological symptoms. On examination, Glasgow Coma Scale (GCS) scored 10/15 (E4V1M5) pupils were equal and reactive. Cerebrospinal fluid (CSF) analysis showed mild elevation of WBCs with no bacterial growth on culture.

**Figure 1:**
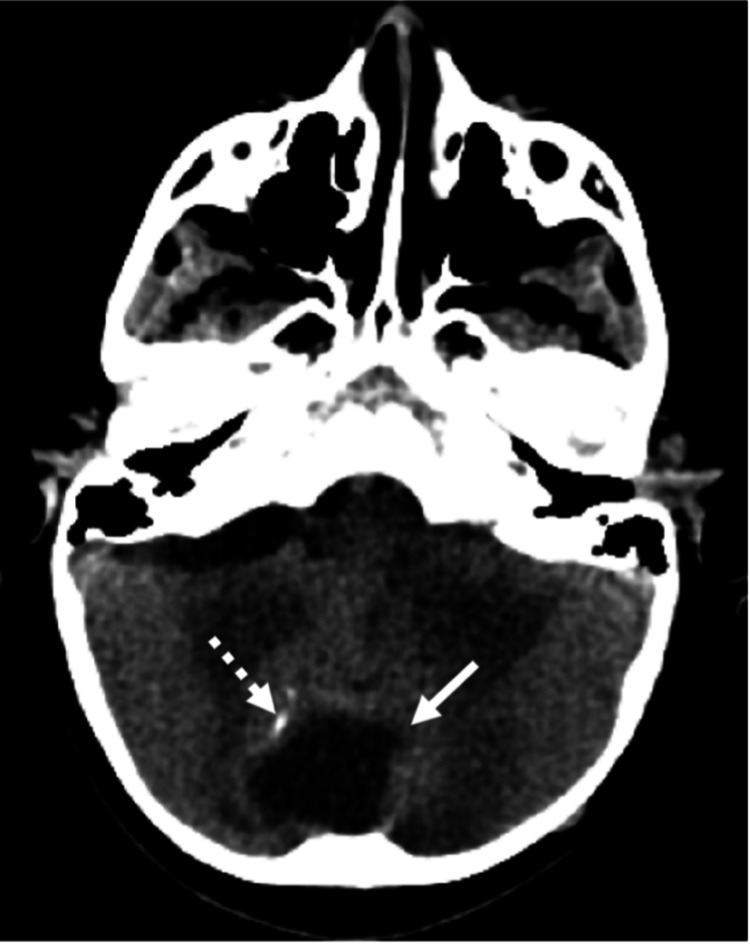
Axial CT (soft tissue window) showing a midline posterior fossa cystic mass (short arrow) with focal peripheral linear calcification (dashed arrow). CT, computed tomography

**Figure 2:**
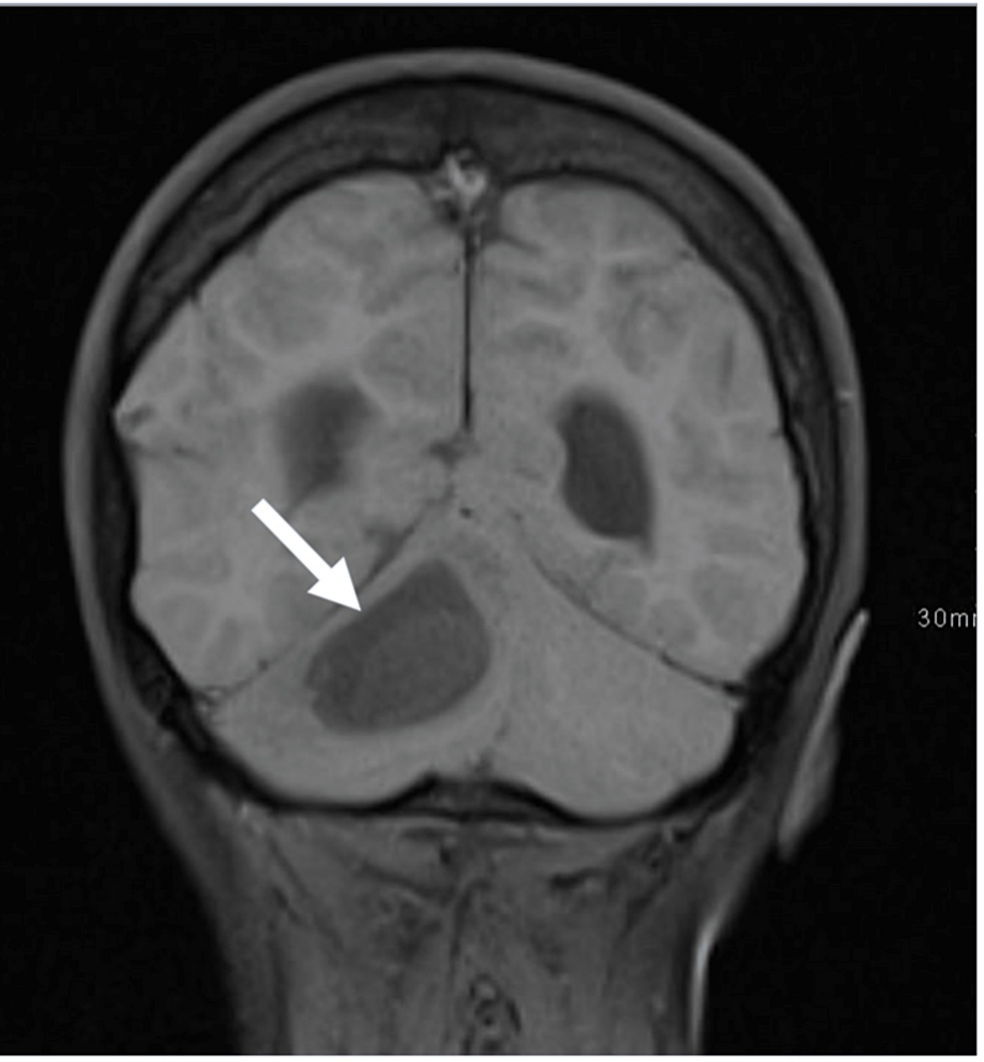
Coronal T1WI showing the heterogeneous low-signal intensity of the cyst (short arrow) in the right cerebellar hemisphere. T1WI, T1-weighted image

**Figure 3:**
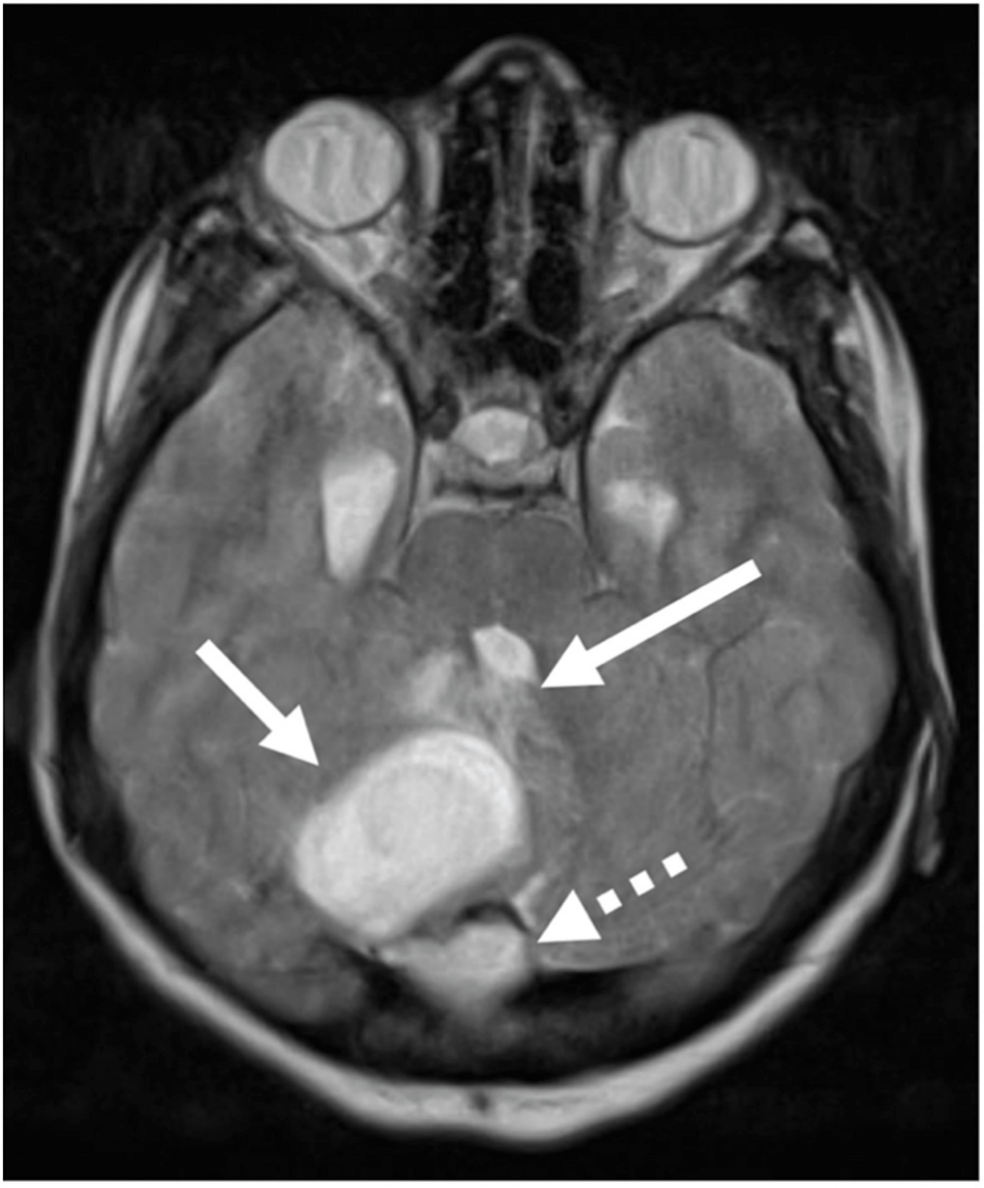
Axial T2WI showing the heterogeneous high-signal intensity of the cyst (short arrow) in the right cerebellar hemisphere, with a bilobar cyst dorsal to the posterior border of the original cyst (dashed arrow); focal surrounding edema and subsequent mass effect in the form of focal effacement of the fourth ventricle (long arrow). T2WI, T2-weighted image

**Figure 4:**
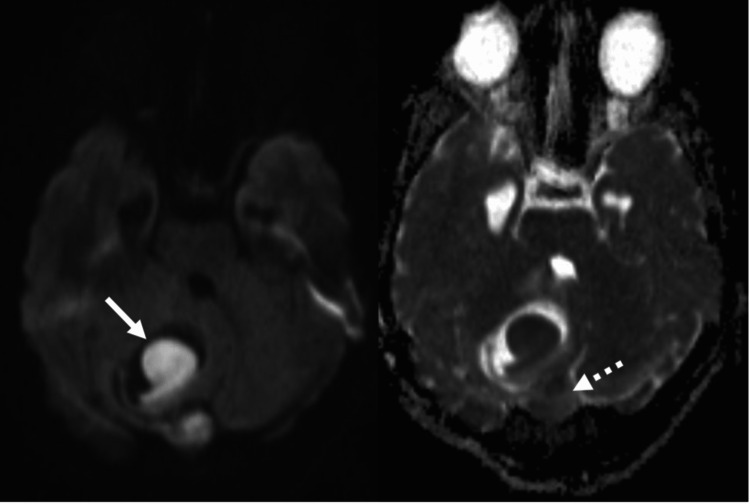
A DWI and ADC map showing a focal central diffusion restriction in the main and daughter cysts, which indicates abscess formation (short and dashed arrows). DWI, diffusion-weighted image; ADC, apparent diffusion coefficient

**Figure 5:**
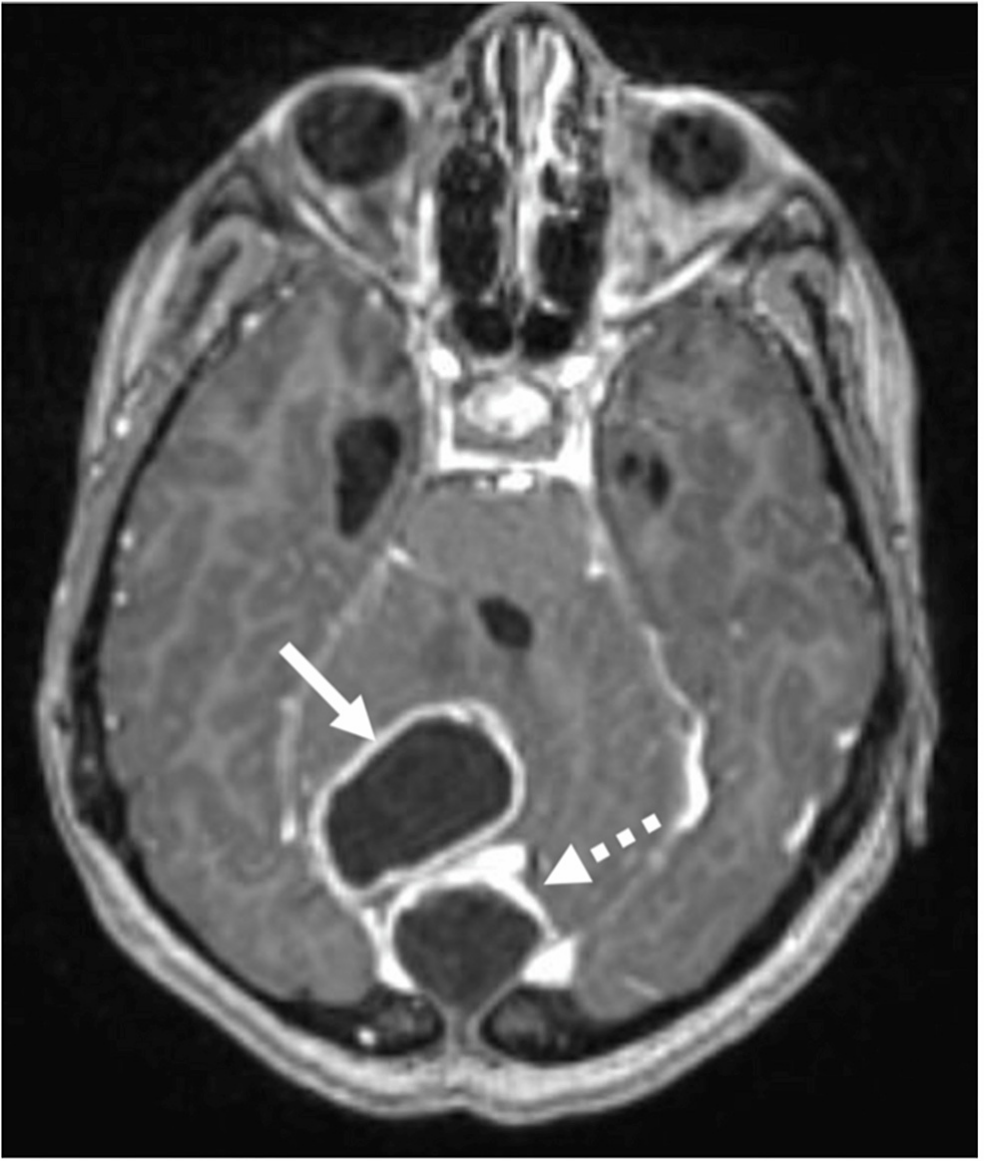
Postcontrast T1WI showing entire smooth peripheral enhancement of the main and daughter cysts (short and dashed arrows). T1WI, T1-weighted image

**Figure 6:**
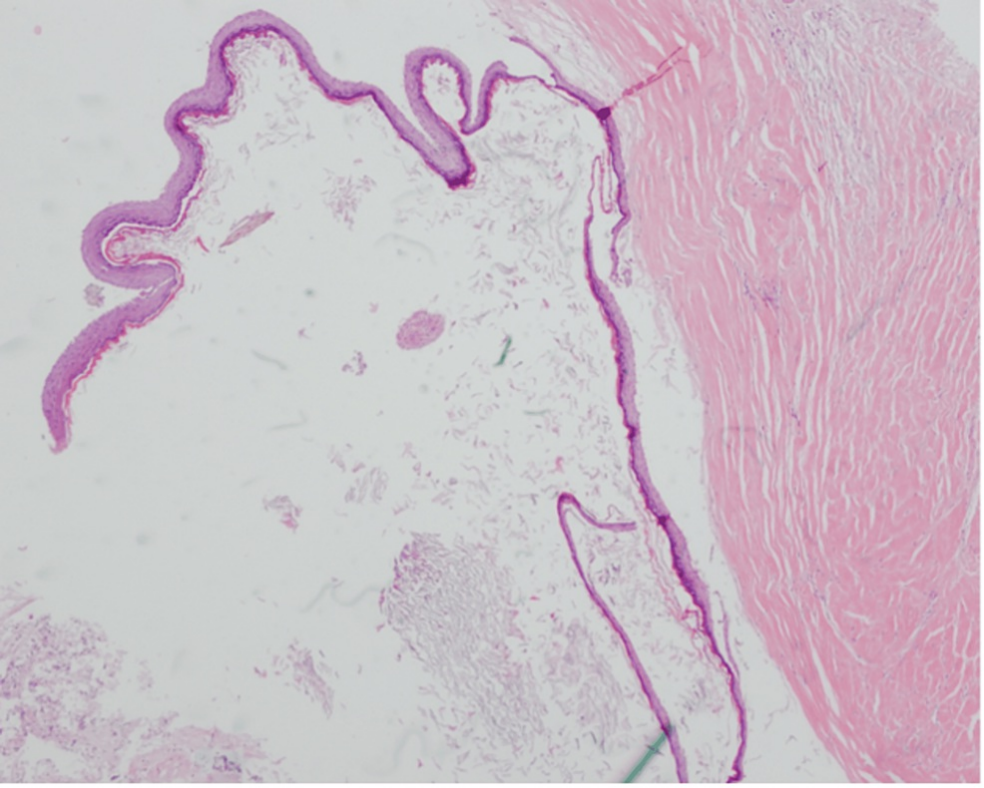
Benign cyst lined by stratified squamous epithelium with a granular cell layer.

Axial computed tomography (CT) of the brain showed a midline posterior fossa cystic mass with focal peripheral linear calcification (Figure 1). Sagittal CT showed a focal bony cortical defect in the occipital bone (Figure 2). Coronal T1WI showed a heterogeneous low-signal intensity of the cyst in the right cerebellar hemisphere (Figure 3). Axial T2-weighted image (T2WI) showed a heterogeneous high-signal intensity of the cyst with a bilobar cyst dorsal to the posterior border of the original cyst (Figure 4). There was a focal central diffusion restriction in the main and daughter cysts, which indicates abscess formation (Figure 5). Postcontrast T1WI showed entire smooth peripheral enhancement of the main and daughter cysts (Figure 6).

**Figure 7:**
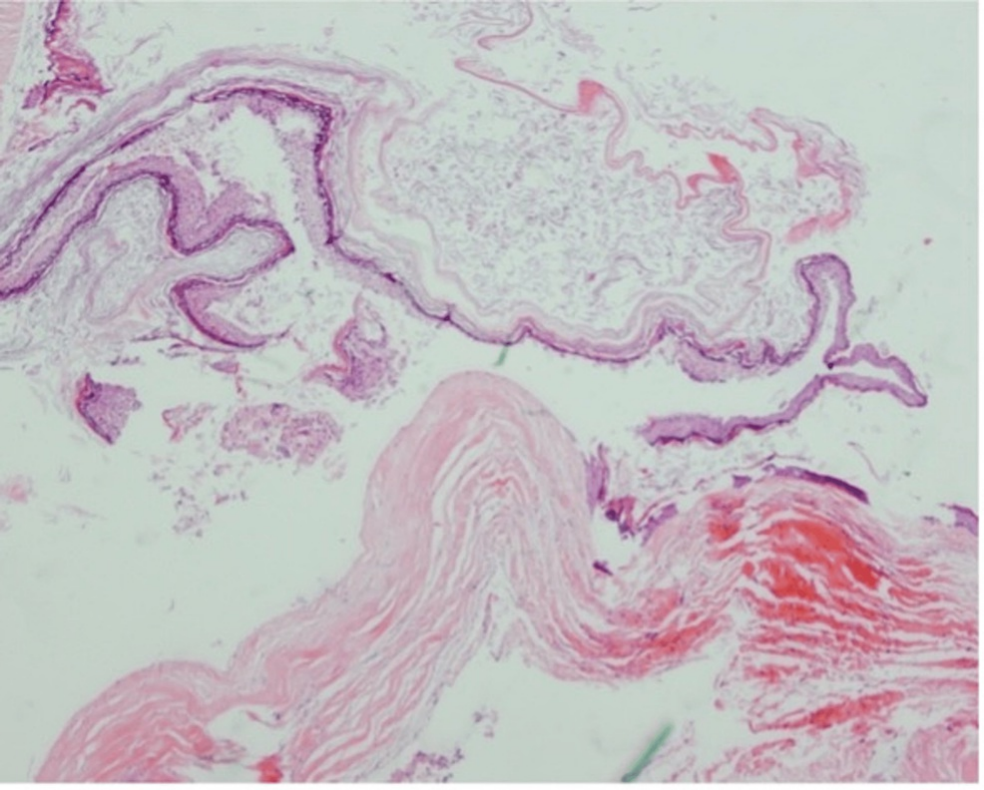
The cyst contains flaky keratin.

**Figure 8:**
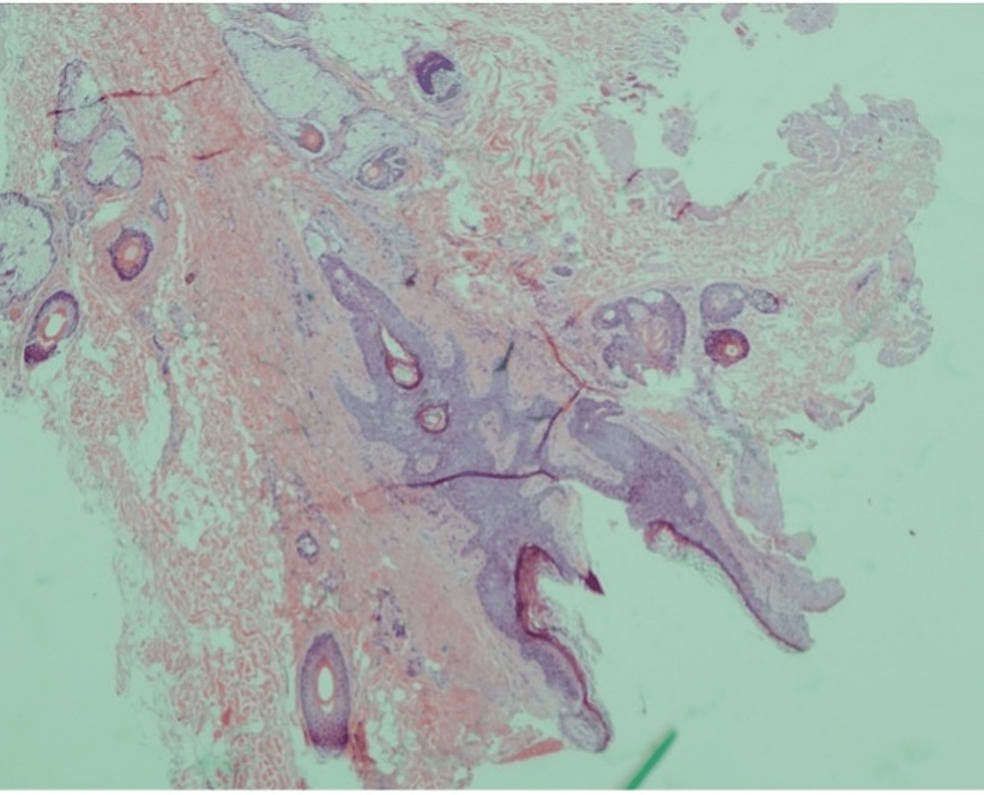
Adnexal structures including hair follicles and sebaceous glands.

Using neuronavigation, the lesion was found to be adherent to the right cerebellum, with a hair-containing sinus attached to it. After dissecting the capsule of the lesion from the surrounding tissue, the specimen was sent to histopathology. Another lesion was found underneath the previously resected lesion, discharging pus, which was similarly dissected and sent for histopathology as well. The histopathological examination of the resected lesion revealed a benign cyst lined by stratified squamous epithelium that contains adnexal structures (Figures 7-9). On her last visit (nine months) after the surgery, she was clinically stable in a wheelchair due to her cerebral palsy.

## Discussion

Origin

Dermoid cysts can develop as a result of various factors, such as the failure of the ectoderm, which is the outermost layer of the embryo, to separate from the underlying neural tube during the third to fifth week of fetal development. Other causes include sequestration or traumatic implantation of skin elements. Congenital dermoid cysts usually arise due to embryonic mishaps during early development stages. Therefore, dermoid cysts tend to appear along the midline, which corresponds to the sites of embryonic fusion. On the other hand, cysts can also develop at any age due to traumatic implantation, which involves the movement of skin elements from the surface to the underlying tissue [2,11].

Classification of intracranial dermoid cysts is based on their relation to the dura, which can be either extradural or intradural. Moreover, they can be further classified based on the presence or absence of an associated sinus. This sinus can either be complete, which means it penetrates the bone, or incomplete, which means it is noncommunicative [12]. A complete sinus refers to a narrow tract lined with epithelium (dermal sinus) that provides a direct communication pathway between the skin and the cyst. This tract contains glandular skin components, and most importantly, it can serve as a pathway for microorganisms to migrate into the cyst, potentially leading to more severe infections [9].

Dermoid cysts located in the infratentorial region are more likely to maintain an attachment to the skin through an occipital bone defect, unlike those situated in the supratentorium. As a result, they are susceptible to infections [13]. Additionally, patients with dermoid cysts in the posterior fossa region and associated dermal sinus are at risk of developing meningitis or focal abscess in the dermoid. In rare cases, daughter abscesses may also form within the cerebellar hemisphere [9]. Nevertheless, cerebellar abscesses, as a complication of a posterior fossa dermoid cyst with dermal sinus, is uncommon with only 16 cases reported in the literature, especially in the absence of a dermal sinus [10,14]. Alternatively, the communication between the cyst and the skin surface may be embryonically disrupted, resulting in a dermal sinus that ends as a blind sac and does not extend into the bone. When an intradural dermoid is linked to an incomplete dermal sinus or an extradural dermoid with any sinus, the likelihood of central nervous system contamination is almost absent [7].

Clinical presentation

Intracranial dermoid cysts are congenital and exhibit gradual growth, marked by the shedding of epithelial cells and secretion of glandular materials within the cyst. Typically, these cysts become symptomatic during the 2nd or 3rd decade of life, and clinical symptoms rarely develop earlier [5,9].

Posterior fossa dermoid cysts may be detected even earlier due to the presence of a visible sinus tract commonly associated with these lesions. The most common presentation is meningitis. They may also rarely present with raised intracranial pressure and mass effect, leading to symptoms such as headaches, seizures, and vomiting. In case of a dermoid cyst rupture, signs of chemical meningitis may be present. Additionally, if the cyst is large enough, it can cause hydrocephalus by obstructing the ventricular system. This scenario is more commonly observed when the dermoid is situated in the posterior fossa or fourth ventricle [2,15]. Our patient's congenital hydrocephalus can be a result of her slow-growing dermoid cyst as Dilnesaw et al. reported a case of posterior fossa dermoid cyst complicated by cerebellar abscess and obstructive hydrocephalus [16]. Although some symptoms may mimic those of posterior fossa tumors, the presence of fever is a distinguishing characteristic of infectious mass lesions in this area [10].

Clinically, a painless tumor or bulge beneath the occipital scalp can sometimes be present. A bone defect may also be tangible, and a sinus entry may be visible. However, the cyst can remain small and asymptomatic until it leads to severe and potentially fatal complications [9,14]. Intracranial dermoid cysts can give rise to severe neurological complications, such as rupture and recurrent meningitis due to their dissemination through CSF (known as aseptic meningitis). They may also become infected, leading to abscess formation. Other complications include hydrocephalus, secondary epilepsy, stroke, or mass effects [17].

Diagnosis of dermoid cysts

Radiological imaging can reveal the presence of a well-defined, midline, unilocular cystic mass, which may be suggestive of a dermoid cyst if it contains true fat or if a fat-fluid level is noticed. Despite their slow growth, dermoid cysts can compress the surrounding structures [2]. In contrast, they are usually not associated with vasogenic edema or contrast enhancement [12].

A CT scan can provide information on the size and exact location of the cyst, as well as detect contraction or discontinuity of the skull bony borders as well as calcification. The CT can also reveal the uniform low density of the cyst content (with attenuation values ranging from -20 to -140 HU), even after contrast administration if the cyst is not infected. However, if the cyst is infected, peripheral enhancement may be observed instead [9]. When imaged with magnetic resonance imaging (MRI), dermoid cysts typically appear hyperintense on T1WI images and show varying degrees of hypo- to hyperintensity on T2WI images, often with nonhomogeneous lesions. They also tend to show moderate restriction on diffusion-weighted images (DWIs). However, if the dermoid cyst has ruptured or if an abscess has formed, hypointensity on T1WI images and marked restriction on DWI images may be observed [14]. Sagittal MRI demonstrates the local occipital defect and typical oblique stalk that connects the dermal sinus and intracranial lesions [9,10].

Management of dermoid cysts

The best optional treatment for dermoid cysts is complete surgical resection, which involves carefully separating the cyst capsule from surrounding neurovascular structures. If performed before infection and abscess formation, total excision of the tumor and sinus tract is optimal. However, complete excision of the cyst may not always be possible due to robust adhesion to vital structures such as venous sinuses. Once the diagnosis is confirmed, surgical intervention should be performed as soon as possible. However, the complete removal of the dermoid cyst may not always be straightforward. That said, if the cyst can be successfully completely resected, recurrence is highly unlikely [8].

The selection of the treatment for cerebellar abscesses depends on various factors, including the patient's clinical status, as well as the size, location, and capsular involvement. Treatment options may include antibiotics, antibiotics with abscess drainage, or in some cases, total removal of the cystic abscess [14]. Dermoid cyst walls are typically thick and completely separated, whereas cerebellar abscess walls are often thin and can be absorbed following external abscess drainage and antibiotic treatment. As a result, it is generally recommended that infected dermoid cysts be completely resected, whereas simple abscesses may be managed with external abscess drainage and antibiotics [10].

Although treatment may be difficult, outcomes are usually excellent, even in patients with large abscesses. If meningitis develops, mortality and morbidity are significantly increased [7].

## Conclusions

If a patient presents with a posterior fossa lesion, along with an occipital bony defect and dermal sinus, there may be a reason to suspect the presence of a congenital dermoid cyst. In such cases, neuroimaging studies are the preferred diagnostic method. Early neurosurgical intervention should be considered to avoid the development of serious complications. Therefore, in cases of new neurological symptoms, abscesses should be considered or screened for. 
